# Screening of hypercortisolism among patients with hypertension: an Italian nationwide survey

**DOI:** 10.1007/s40618-024-02387-2

**Published:** 2024-06-24

**Authors:** G. Di Dalmazi, J. Goi, J. Burrello, L. Tucci, A. F. G. Cicero, C. Mancusi, E. Coletti Moia, G. Iaccarino, C. Borghi, M. L. Muiesan, C. Ferri, P. Mulatero

**Affiliations:** 1 Division of Endocrinology and Diabetes Prevention and Care, IRCCS Azienda Ospedaliero-Universitaria di Bologna, Bologna, Italy; 2 Department of Medical and Surgical Sciences (DIMEC), Alma Mater Studiorum University of Bologna, Bologna, Italy; 3 Division of Internal Medicine and Hypertension, Department of Medical Sciences, University of Torino, Turin, Italy; 4 Hypertension and Cardiovascular Risk Research Unit, Medical and Surgery Sciences Department, Alma Mater Studiorum University of Bologna, Bologna, Italy; 5 Department of Advanced Biomedical Sciences, Federico II University of Naples, Via S. Pansini 5, 80131 Naples, Italy; 6 ARCA (Associazioni Regionali Cardiologi Ambulatoriali) sezione Piemonte, Turin, Italy; 7 Department of Clinical and Experimental Sciences, University of Brescia, Brescia, Italy; 8 Department of Life, Health and Environmental Sciences University of L’Aquila, L’Aquila, Italy

**Keywords:** Hypertension, Cushing, MACS, Hypercortisolism, Survey

## Abstract

**Purpose:**

Screening of Cushing Syndrome (CS) and Mild Autonomous Cortisol Secretion (MACS) in hypertensive patients is crucial for proper treatment. The aim of the study was to investigate screening and management of hypercortisolism among patients with hypertension in Italy.

**Methods:**

A 10 item-questionnaire was delivered to referral centres of European and Italian Society of Hypertension (ESH and SIIA) in a nationwide survey. Data were analyzed according to type of centre (excellence *vs* non-excellence), geographical area, and medical specialty.

**Results:**

Within 14 Italian regions, 82 centres (30% excellence, 78.790 patients during the last year, average 600 patients/year) participated to the survey. Internal medicine (44%) and cardiology (31%) were the most prevalent medical specialty. CS and MACS were diagnosed in 313 and 490 patients during the previous 5 years. The highest number of diagnoses was reported by internal medicine and excellence centres. Screening for hypercortisolism was reported by 77% in the presence of specific features of CS, 61% in resistant hypertension, and 38% in patients with adrenal mass. Among screening tests, the 24 h urinary free cortisol was the most used (66%), followed by morning cortisol and ACTH (54%), 1 mg-dexamethasone suppression test (49%), adrenal CT or MRI scans (12%), and late night salivary cortisol (11%). Awareness of referral centres with expertise in management of CS was reported by 67% of the participants, which reduced to 44% among non-excellence centres.

**Conclusions:**

Current screening of hypercortisolism among hypertensive patients is unsatisfactory. Strategies tailored to different medical specialties and type of centres should be conceived.

**Supplementary Information:**

The online version contains supplementary material available at 10.1007/s40618-024-02387-2.

## Background

Cushing’s syndrome and Mild Autonomous Cortisol Secretion (MACS, formerly known as subclinical Cushing’s syndrome or subclinical hypercortisolism) are different aspects of the spectrum of endogenous hypercortisolism. Cushing’s syndrome is a rare disease (2–3 per million people) diagnosed in patients with cortisol excess associated with a typical clinical picture characterized by muscle weakness, *striae rubrae*, facial plethora, buffalo hump, and supraclavicular adiposity [1, 2]. On the other side, MACS is defined by endogenous hypercortisolism in the absence of catabolic signs of Cushing’s syndrome, representing the most frequent hormonal alteration in patients with adrenal incidentalomas [2–4].

Despite the differences in phenotypic expression, Cushing’s syndrome and MACS have both been associated with an increased risk of cardiovascular events and mortality, mostly mediated by hypertension. In Cushing’s syndrome, hypertension occurs in up to 93% of patients and can be associated with left ventricular hypertrophy, vascular atherosclerosis, and thrombosis diathesis, leading to an increased overall mortality due to venous thromboembolism, myocardial infarction, and stroke [5, 6]. Similarly, hypertension is the most frequent comorbidity associated with MACS (64% of the patients), and resistant hypertension has been linked to co-secretion of cortisol and corticosterone [7]. MACS have been identified as an independent risk factor for major cardiovascular events, atrial fibrillation, and increased all-cause and cardiovascular-specific mortality, with the highest risk in younger women [7–11]. Among hypertensive patients, Cushing’s syndrome has been diagnosed in up to 1%, whereas MACS in up to 27% of patients with resistant hypertension [12, 13].

Several previous studies have successfully demonstrated that control of hypercortisolism is key in patients with Cushing’s syndrome, through improvement or cure of hypertension and reduction of the cardiovascular risk, which however remains high even years after remission [14–16]. Even though the data on MACS are less solid, several evidences points toward a beneficial effect of adrenalectomy on the control of hypertension and cardiovascular risk factors [17, 18].

Nevertheless, Cushing’s syndrome is still burdened by a large diagnostic delay of up to 34 months after onset of symptoms, despite the typical clinical presentation, whereas MACS has been considered a condition without clear cardiometabolic implications until recently [19, 20]. Indeed, an early detection of endogenous hypercortisolism is of utmost importance to address the patients to proper treatments, especially among hypertensive patients.

The aim of this study was to investigate the current rate of screening and management of hypercortisolism among hypertensive patients evaluated at referral centres for hypertension in Italy, belonging to the European Society of Hypertension (ESH) and the Italian Society of Hypertension (SIIA).

## Methods

We designed a questionnaire for physicians treating patients with hypertension, to investigate the current screening methods for hypercortisolism. The questionnaire was composed of 10 items and is provided in Supplementary Table 1. We enrolled centres belonging to the ESH and the SIIA in 14 Italian regions, to which the questionnaire was sent via institutional communication channels of the Societies, with the help of the regional sections and the ARCA (Associazioni Regionali Cardiologi Ambulatoriali) Piemonte. Participating centres were divided into excellence centres (centres certified by the Societies as reference centre for care of hypertension) and non-excellence centres.

Data were presented as descriptive statistics for the whole cohort of participating centres. Data were also analyzed after stratification by geographical area (North Italy *vs* Centre-South Italy), prevalent specialty of the responding physicians (Internal Medicine *vs* Cardiology *vs* other), and type of the centres (excellence *vs* non-excellence centres).

Statistical analysis was performed by IBM SPSS Statistics 26 (IBM Corp, Armonk, NY) and GraphPad Prism 9.0 (GraphPad, La Jolla, CA). Data are presented as median and interquartile range, or frequencies, as appropriated. Comparison among groups was performed by Chi-square or Fisher’s tests for categorical variables, and with Kruskal–Wallis or Mann–Whitney tests for scalar variables. *P* < 0.05 was considered statistically significant.

## Results

**Fig. 1:**
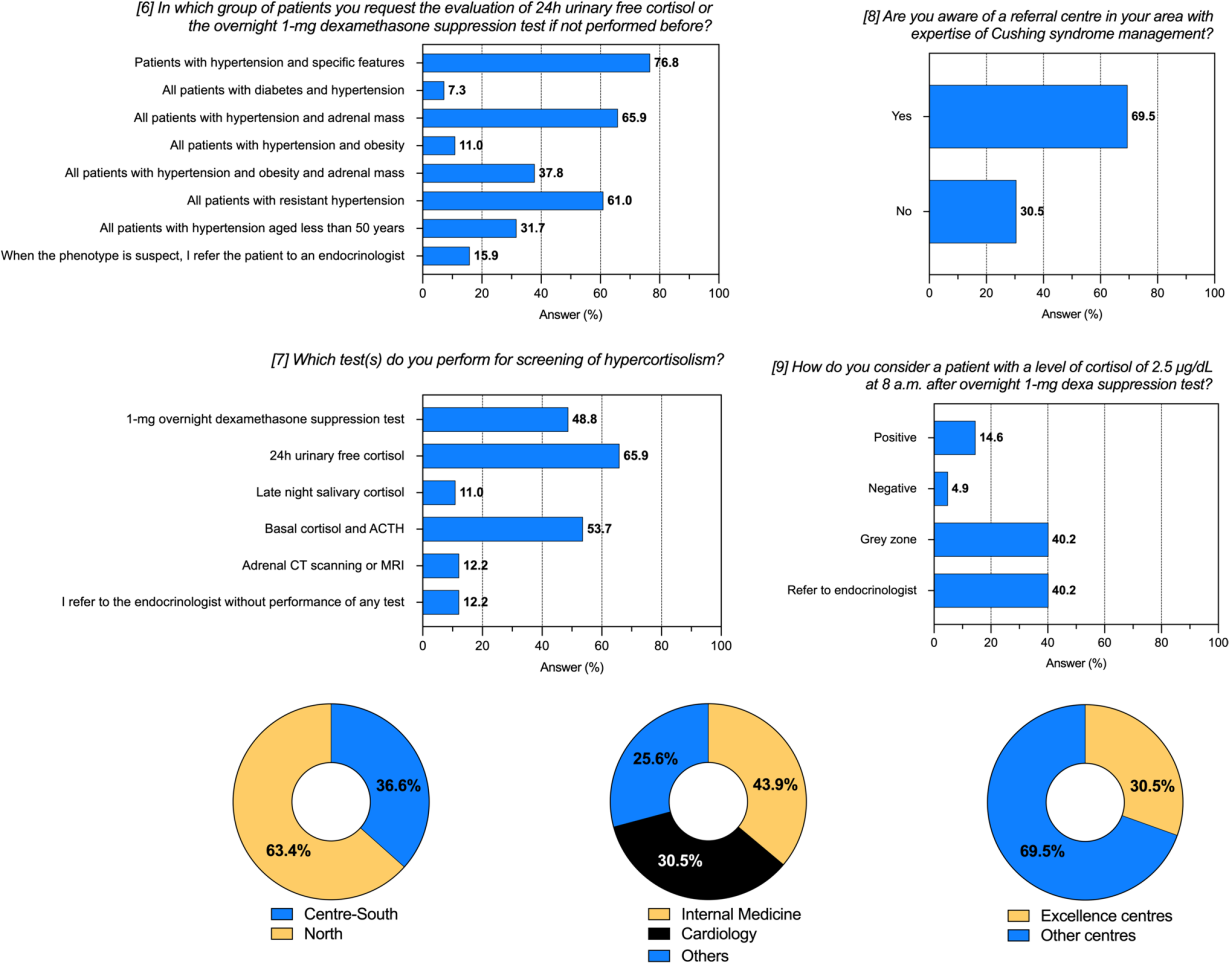
Responses to questions 6–9 of the questionnaire. The histograms report frequencies (%), whereas pie charts centre distribution for geographical areas, prevalent medical specialty, and type of centre. See also Tables 1, 2

A total of 82 centres were involved, as shown in Table 1. Most of the responding centres were located in the north of Italy (63%). About one third (30%) was labelled as excellence centre. The most prevalent medical specialty was internal medicine (44%), followed by cardiology (31%), while endocrinologists represented 10% (n = 8 centres) of the total responders (Fig. 1).

**Table 1:** Enrolled centres and characteristics

Variable	Centres (n = 82)
Average number of referred patient in 1 year (n)	600 [300; 1500]
Patients evaluated as first visit (n)	175 [100; 313]
Patients with resistant hypertension (%)	10.0 [5.0; 25.0]
Cases of Cushing syndrome in the last 5 years (average n per center)	1 [0; 5]
Pituitary (%)	25.0 [0.0; 50.0]
Adrenal (%)	75.0 [40.0; 100.0]
Total (Absolute number in 5 years)	313 (from 50 centres)
Cases of MACS in the last 5 years (average n per center)	1 [0; 4]
Total (Absolute number in 5 years)	490 (from 41 centres)
Geographical areas:	
North of Italy (n; %)	52 (63.4%)
Centre-South of Italy (n; %)	30 (36.6%)
Prevalent specialty:	
Internal Medicine (n; %)	36 (43.9%)
Cardiology (n; %)	25 (30.5%)
Others* (n; %)	21 (25.6%)
Excellence centres (n; %)	25 (30.5%)

The table reports characteristics of the enrolled centres and number of referred patients with Cushing syndrome or MACS. Data are reported as median [interquartile range] or absolute number and frequencies, as appropriate.

*Others: endocrinology, nephrology, geriatrics

The total number of patients evaluated by all centres during the year before the date of response was 78.790, of which 21.890 as first evaluations. The average number of patients referred per year to each centre was 600 (range 300–1500), with 10% (range 5–25%) of subjects having resistant hypertension. The number of patients diagnosed with Cushing’s syndrome and MACS during the last five years was 313 and 490, respectively, with a median of 1 case for both diseases. The proportion of centres that did not report diagnoses of Cushing’s syndrome and MACS during the last five years was 39% (32/82) and 50% (41/82), respectively. The analysis of those parameters according to geographical area did not highlight significant differences, whereas when considering the prevalent medical specialty, a higher number of patients diagnosed with Cushing’s syndrome during the last 5 years was recorded by internal medicine specialists (*P* = 0.009), when compared to other groups (Supplementary Table 2). Excellence centres had a higher average number of patients per year than those labeled as non-excellence and a higher number of patients diagnosed with Cushing’s syndrome and MACS.

Table 2 shows the results of the questions on the diagnosis and management of hypercortisolism. Among all, 63/82 (77%) of the centres would perform a hormonal evaluation in the presence of hypertension and specific features of Cushing’s syndrome, whereas 50/82 (61%) would do so in the presence of resistant hypertension. Importantly, only 31/82 (38%) of the centres would ask for hormonal evaluation in the case of hypertension and adrenal mass. When the suspicion of hypercortisolism leads to hormonal assessment, the 24 h urinary free cortisol (24 h-UFC) is the most frequent test (66%), followed by basal (i.e. morning) cortisol and adrenocorticotrophic hormone (ACTH) (54%), 1 mg-dexamethasone suppression test (DST) (49%), and late night salivary cortisol (LNSC) (11%). Remarkably, adrenal computerized tomography (CT) or magnetic resonance imaging (MRI) was considered as a screening tool for hypercortisolism by 10/82 centres (12%). Most centres (57/82, 67%) were aware of a referral centre with expertise in the management of Cushing’s syndrome in their area.

**Table 2:** Management of hypercortisolism and Cushing’s syndrome

All Centres (n = 82)	
*(*6) In which group of patients, you request the evaluation of 24 h urinary free cortisol or the overnight 1-mg dexamethasone suppression test if not performed before?	
(a) Patients with hypertension and specific features (buffalo hump, moon facies, purple reddish striae)	63 (76.8)
(b) All patients with diabetes and hypertension	6 (7.3)
(c) All patients with hypertension and adrenal mass	55 (65.9)
(d) All patients with hypertension and obesity	9 (11.0)
(e) All patients with hypertension and obesity and adrenal mass	31 (37.8)
(f) All patients with resistant hypertension	50 (61.0)
(g) All patients with hypertension aged less than 50 years	26 (31.7)
(h) When the phenotype is suspect, I refer the patient to an endocrinologist	13 (15.9)
(7) Which test(s) do you perform for screening of hypercortisolism?	
(a) 1-mg overnight dexamethasone suppression test	40 (48.8)
(b) 24 h urinary free cortisol	54 (65.9)
(c) Late night salivary cortisol	9 (11.0)
(d) Basal cortisol and ACTH	44 (53.7)
(e) Adrenal CT scanning or MRI	10 (12.2)
(f) I refer to the endocrinologist without performance of any test	10 (12.2)
(8) Are you aware of a referral centre in your area with expertise of Cushing syndrome management?	
(a) Yes	57 (69.5)
(b) No	25 (30.5)
(9) How do you consider a patient with a level of cortisol of 2.5 μg/dL at 8 a.m. after overnight 1-mg dexamethasone suppression test?	
(a) Positive	12 (14.6)
(b) Negative	4 (4.9)
(c) Grey zone, I request further tests	33 (40.2)
(d) I refer to an endocrinologist	33 (40.2)

Responses to questions 6-to-9 of the questionnaire. Data are reported as absolute numbers and frequencies, as appropriate

**Fig. 2:**
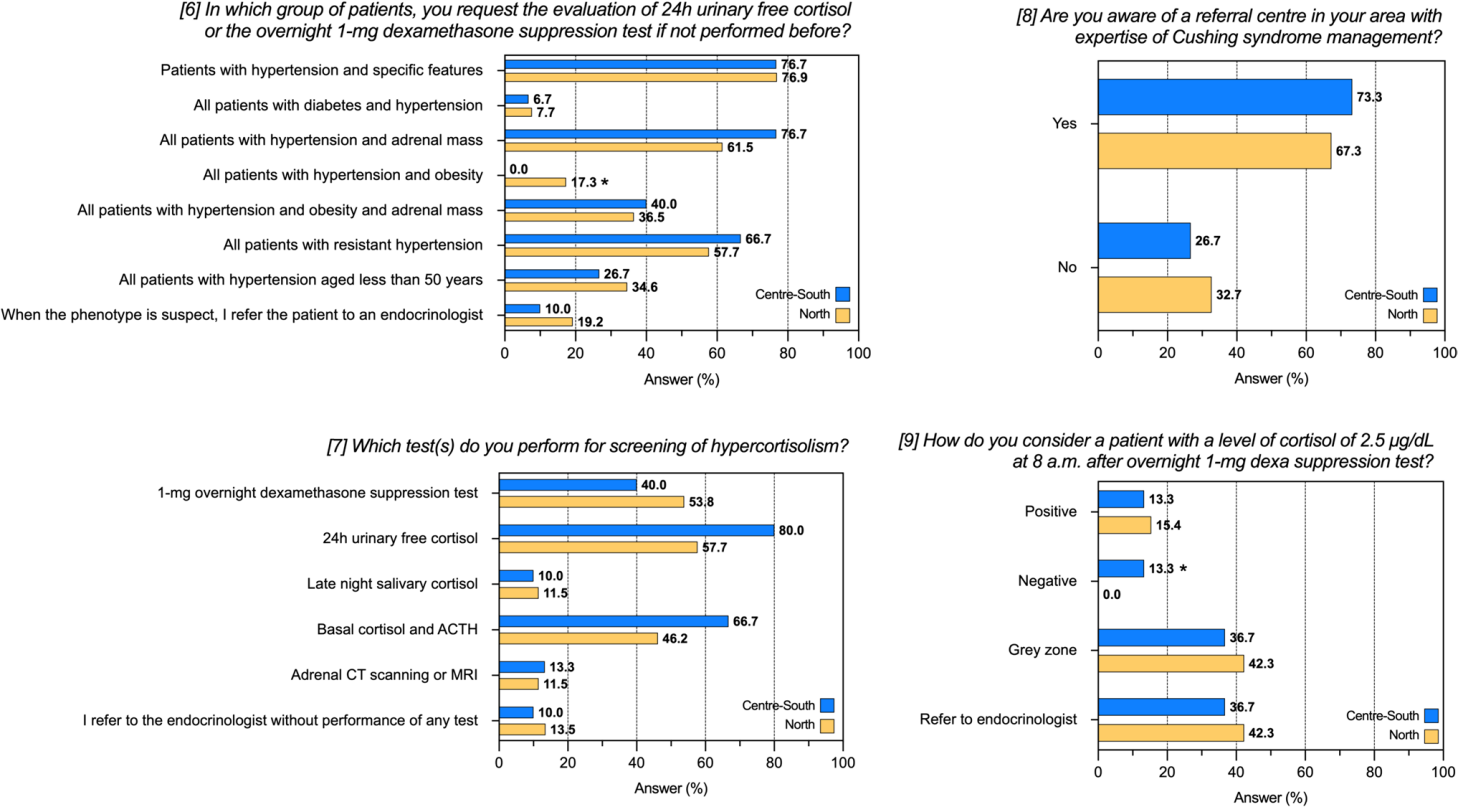
Responses to questions 6–9 of the questionnaire after stratification for geographical areas. Data are reported as absolute numbers and frequencies, as appropriate. *P-*value < 0.05 were considered significant and highlighted in bold. See also Table S3. *P < 0.05

**Fig. 3:**
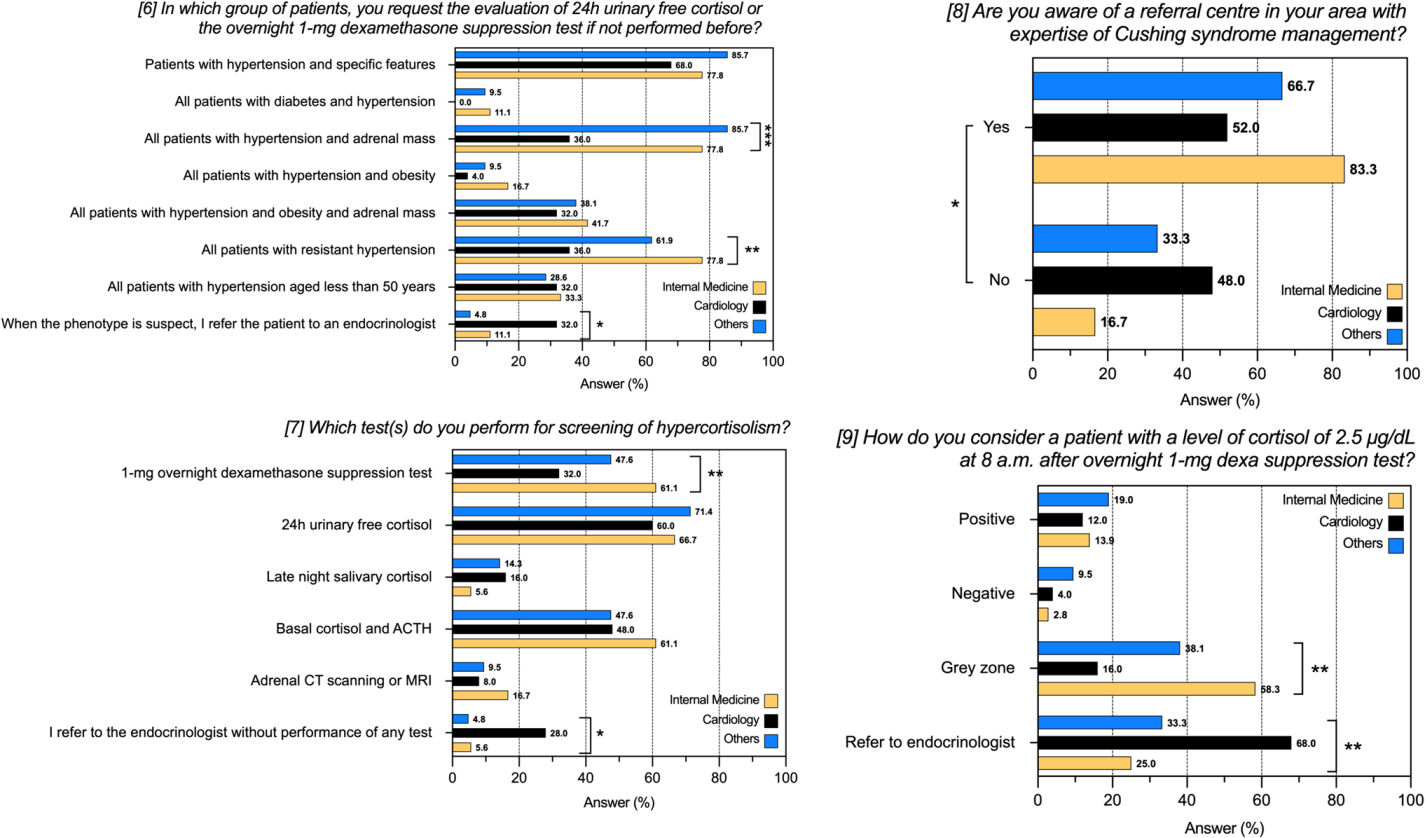
Responses to questions 6–9 of the questionnaire after stratification for prevalent medical specialty. Data are reported as absolute numbers and frequencies, as appropriate. *P-*value < 0.05 were considered significant and highlighted in bold. See also Table 3. *P < 0.05; **P < 0.01; ***P < 0.001

The analysis by geographical area did not highlight significant differences, except that no centres in the center-south of Italy would request the evaluation for hypercortisolism in patients with hypertension and obesity (Supplementary Table 3 and Fig. 2). The analysis according to the prevalent specialty of responding physicians (Table 3 and Fig. 3) revealed a lower proportion of screening among cardiologists than others for patients with hypertension and adrenal mass (*P* < 0.001) and resistant hypertension (*P* = 0.004), as well as a higher rate of referral to the endocrinologists (*P* = 0.034). The 1-mg DST was the most frequent test requested by internal medicine centres (61%), when compared to cardiology (32%) and other (48%) centres (*P* = 0.003). No significant differences were detected for the use of other tests, confirming the data of the whole cohort. A higher proportion of centres not aware of a reference centre for Cushing’s syndrome was detected among cardiologists (48%) (*P* = 0.031).

**Table 3:** Management of hypercortisolism and Cushing’s syndrome according to prevalent medical specialty

Prevalent Specialty (n of centers)	Internal Medicine (n = 36)	Cardiology (n = 25)	Others* (n = 21)	*P-*value
(6) In which group of patients, you request the evaluation of 24 h urinary free cortisol or the overnight 1-mg dexamethasone suppression test if not performed before?				
(a) Patients with hypertension and specific features (buffalo hump, moon facies, purple reddish striae)	28 (77.8)	17 (68.0)	18 (85.7)	0.360
(b) All patients with diabetes and hypertension	4 (11.1)	0 (0.0)	2 (9.5)	0.228
(c) All patients with hypertension and adrenal mass	28 (77.8)	9 (36.0)	18 (85.7)	** < 0.001**
(d) All patients with hypertension and obesity	6 (16.7)	1 (4.0)	2 (9.5)	0.322
(e) All patients with hypertension and obesity and adrenal mass	15 (41.7)	8 (32.0)	8 (38.1)	0.805
(f) All patients with resistant hypertension	28 (77.8)	9 (36.0)	13 (61.9)	**0.004**
(g) All patients with hypertension aged less than 50 years	12 (33.3)	8 (32.0)	6 (28.6)	0.932
(h) When the phenotype is suspect, I refer the patient to an endocrinologist	4 (11.1)	8 (32.0)	1 (4.8)	**0.034**
(7) Which test(s) do you perform for screening of hypercortisolism?				
(a) 1-mg overnight dexamethasone suppression test	22 (61.1)	8 (32.0)	10 (47.6)	**0.003**
(b) 24 h urinary free cortisol	24 (66.7)	15 (60.0)	15 (71.4)	0.712
(c) Late night salivary cortisol	2 (5.6)	4 (16.0)	3 (14.3)	0.358
(d) Basal cortisol and ACTH	22 (61.1)	12 (48.0)	10 (47.6)	0.052
(e) Adrenal CT scanning or MRI	6 (16.7)	2 (8.0)	2 (9.5)	0.687
(f) I refer to the endocrinologist without performance of any test	2 (5.6)	7 (28.0)	1 (4.8)	**0.020**
(8) Are you aware of a referral centre in your area with expertise of Cushing syndrome management?				
(a) Yes	30 (83.3)	13 (52.0)	14 (66.7)	**0.031**
(b) No	6 (16.7)	12 (48.0)	7 (33.3)	
(9) How do you consider a patient with a level of cortisol of 2.5 μg/dL at 8 a.m. after overnight 1-mg dexamethasone suppression test?				
(a) Positive	5 (13.9)	3 (12.0)	4 (19.0)	0.852
(b) Negative	1 (2.8)	1 (4.0)	2 (9.5)	0.681
(c) Grey zone, I request further tests	21 (58.3)	4 (16.0)	8 (38.1)	**0.004**
(d) I refer to an endocrinologist	9 (25.0)	17 (68.0)	7 (33.3)	**0.003**

Responses to questions 6–9 of the questionnaire after stratification for prevalent medical specialty. Data are reported as absolute numbers and frequencies, as appropriate. *Others: endocrinology, nephrology, geriatrics. *P-*value < 0.05 were considered significant and highlighted in bold

The sub analysis by prevalent medical specialty was performed also considering the 8 centres of endocrinology as a separate group (data not shown). All the endocrinological centres would screen for hypercortisolism in the presence of patients with hypertension and adrenal mass, as well as patients with resistant hypertension (*P* < 0.001 for both). A significant higher proportion of endocrinology centres would prefer the 1 mg DST and LNSC as screening tools for hypercortisolism (*P* = 0.004 and *P* = 0.032, respectively).

**Fig. 4:**
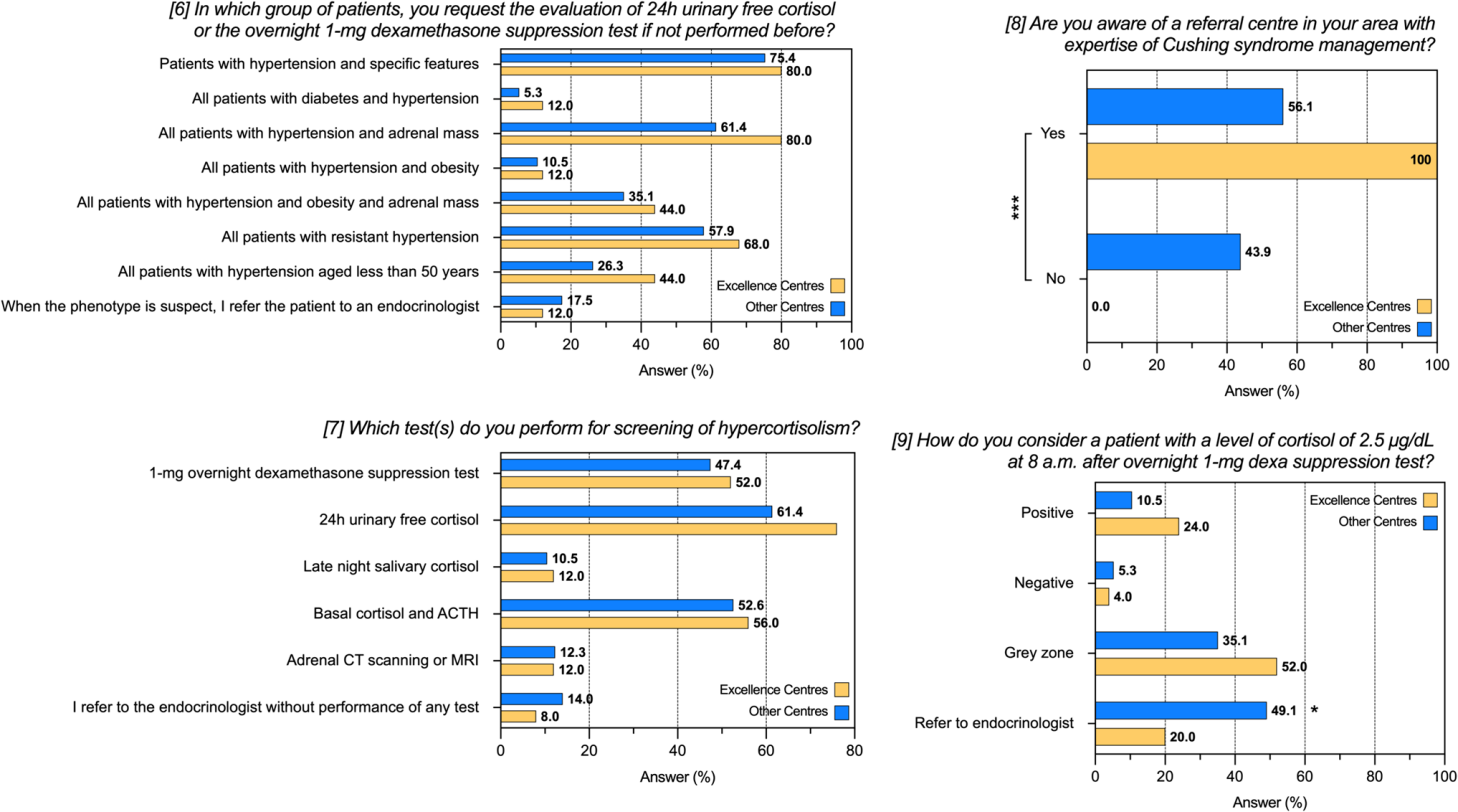
Responses to questions 6–9 of the questionnaire after stratification according to type of centre. Data are reported as absolute numbers and frequencies, as appropriate. *P-*value < 0.05 were considered significant and highlighted in bold. See also Table S4. *P < 0.05; ***P < 0.001

No significant differences in the categories of patients to be screened for hypercortisolism nor in the preferred screening tests were detected between excellence *vs* non-excellence centres. Almost half of non-excellence centres (25/57, 44%) was not aware of a referral centre with expertise in the management of Cushing’s syndrome in their area (*P* < 0.001 *vs* excellence centres, 0%) (Supplementary Table 4 and Fig. 4). Among those, 40% of the centres (n = 10) would refer a patient with a cortisol after 1 mg DST > 2.5 mcg/dL to an endocrinologist. Similarly, the proportion of non-excellence centres that are aware of a referral centre for Cushing’s syndrome that would refer a patient with such a hormonal profile to an endocrinologist was 56% (n = 18).

## Discussion

This study provides the results of a survey on the diagnosis and management of hypercortisolism, among Italian centres affiliated to SIIA and ESH managing patients with hypertension. The survey covered a large proportion of Italian territory, by involving 82 centres in 14 regions with almost 80.000 patients, representing a large population in a real-life setting (the proportion of resistant hypertension was in line with the literature) [21, 22].

The survey highlighted that screening of hypercortisolism still remains an issue across different medical specialties. Importantly, roughly one third of the centres would not screen for hypercortisolism even in the presence of hypertension and specific features of Cushing’s syndrome. This holds true independently of geographical distribution of the centre, prevalent medical specialty or type of the centre (excellence *vs* non-excellence). An impressive high proportion of underscreening has also been identified in patients with hypertension and adrenal mass (60% of the centres). Notably, young hypertensive patients < 50 years are not undergoing screening for hypercortisolism in the majority of the centres (roughly 70%), with no difference among medical specialists. A large rate of underscreening of hypercortisolism was also identified in patients with resistant hypertension (40% of the centres), with an even higher proportion of underscreening among cardiologists. Even if the data collection was not planned for calculating the prevalence of cases of Cushing’s syndrome and MACS among hypertensive patients, some estimates can be extrapolated. When only first evaluations of patients were considered (since the likelihood of diagnosing hypercortisolism might be higher in this category of patients), the prevalence of new diagnoses is 0.3% (average of 63 cases/21.890 patients/year) for Cushing’s syndrome and 0.5% (average of 98 cases/21.890 patients/year) for MACS. Those numbers are much lower than expected, since the prevalence of Cushing’ s syndrome and MACS among hypertensive patients was up to 2% and 1–8%, respectively, in previous studies [23–25]. The proportion of patients receiving a diagnosis of hypercortisolism among the total cohort of patients evaluated by the centres are even lower (0.08% for Cushing’s syndrome and 0.12% for MACS).

According to the current guidelines, patients with specific features of hypercortisolism, like easy bruising, facial plethora, *striae rubrae*, myopathy or weakness in proximal muscles and unusual features for age, should all be screened for Cushing’s syndrome [1, 2, 16, 26]. The presence of these features, in combination with other signs or symptoms potentially related to hypercortisolism, even if less specific, increase the pre-test probability of Cushing’s syndrome, and should prompt a rapid screening for hypercortisolism. In particular, some specific characteristics associated with hypercortisolism has been recognized among hypertensive patients. In a recent consensus published by the working group on secondary hypertension of the ESH, the screening of Cushing’s syndrome should be performed in young patients (< 40 years) with at least grade 2 hypertension, children with hypertension, subjects with resistant hypertension independently of age, and patients with non-dipping blood pressure profile [16]. Moreover, the presence of hypokalemia should also prompt the screening for hypercortisolism. Furthermore, the analysis of white blood cells might also be helpful in the screening of Cushing’syndrome, according to recent data [27, 28]. Patients with adrenal incidentalomas should be screened all for MACS, independently of the presence of specific features of Cushing’s syndrome. MACS is not associated with catabolic signs of Cushing’s syndrome, by definition. However, hypertension is frequently associated with this disorder, with a pooled relative risk of 1.24 (95% confidence interval 1.16–1.32) in 31 studies, when MACS is defined by cortisol levels after 1 mg-DST > 1.8 µg/dL) [29].

According to the results of this survey, when the screening of hypercortisolism is planned, among the three screening tests for Cushing’s syndrome, 24 h-UFC is the most frequently used, followed by 1 mg DST and LNSC, the latter performed almost exclusively by endocrinologists. 24 h-UFC is being largely used for screening of Cushing’s syndrome, since it reflects the 24 h urinary output of free cortisol, with a sensitivity of 94% and a specificity of 93% [30]. Nevertheless, incomplete urine collection and impaired GFR may lead to false negative results. Therefore, confirmation by a repeated urinary free cortisol assay is suggested by the guidelines, even though intra-individual variations of cortisol excretion is an issue that should be taken into account when interpreting the results [1, 2, 16, 31]. The 1 mg DST has shown a high sensitivity (99%) with a specificity of 91% for diagnosis of Cushing’s syndrome [30]. Given the high sensitivity at the cortisol cut off of 1.8 µg/dL, the 1 mg DST is appropriate for screening purposes, after having excluded potential sources of bias (incomplete or lack of ingestion of dexamethasone by the patient, changes in cortisol binding globulin or albumin, and drugs altering the CYP3A4 activity) [31]. Collection of LNSC is a simple and non-invasive test that can be performed at home by the patients, with sensitivity and specificity for the diagnosis of Cushing’s syndrome > 90% [32]. The reliability of this test may be reduced by night shifts or altered sleep–wake cycles, blood contamination of the sample, and large intra-individual variability, therefore repeated sampling is suggested by the guidelines [1, 2]. Considering the performance and the pitfalls of each test, a larger use of LNSC is desirable as a valid method for improving the low screening rate of Cushing’s syndrome among hypertensive patients.

Although 24 h-UFC and LNSC are appropriate for screening Cushing’s syndrome, they are not valid methods for diagnosing MACS, because those tests may be often normal in this condition [2]. According to the most recent guidelines, MACS should be diagnosed by using 1 mg DST as a single test, by using a revised cut off for cortisol of 1.8 µg/dL, without further grading of severity based on additional hormonal tests [2].

It is noteworthy that an important proportion of centres use inappropriate tool for screening hypercortisolism, like morning ACTH and cortisol (roughly half of the centres) and CT or MRI scan (12% of responders). Baseline ACTH and cortisol should not be considered as screening tests for hypercortisolism, because ACTH may be within the normal range in pituitary Cushing’s syndrome and morning cortisol may not be elevated. Morning ACTH is a useful test for subtyping Cushing’s syndrome, once the diagnosis has been established by appropriate testing [1, 2]. The use of imaging as a screening tool rise the possibility to detect incidentalomas, which are very common and are not necessarily associated with hypercortisolism. In particular, adrenal incidentalomas are discovered in 3–4.2% of patients undergoing abdominal imaging and pituitary incidentalomas in 15–21 per 100.000 in the general population (16–36% of pituitary adenomas) [3, 33].

The results of the survey also highlight an inappropriate referral to proper centres of patients with hypercortisolism. Indeed, half of the non-excellence centres are not aware of a centre with expertise in Cushing’s syndrome (a situation dramatically different from the excellence centres, with 100% of awareness) and less than half of them would refer a patient with hypercortisolism to an endocrinologist. These data point toward an insufficient network of collaboration between non-excellence centres managing patients with hypertension and tertiary endocrinological centres, leading to a referral of patients with hypercortisolism that is insufficient (60% of the patients are not addressed to an expert endocrinological centre) and inappropriate (the remaining patients are referred to endocrinologists who are likely not expert in managing Cushing’s syndrome).

Therefore, the survey highlights an unsatisfactory screening rate for Cushing’s syndrome among hypertensive patients, which may explain in part the reason for the delay in the diagnosis of this condition [19]. The prompt recognition of Cushing’s syndrome is pivotal, to address the patient to a proper treatment to control the hormonal excess and manage hypertension, since the latter has been associated independently with increased mortality and duration and severity of hypercortisolism [5, 34–36]. Even though the association between hypertension and MACS is based mostly on retrospective and observational data, two randomized controlled trials have demonstrated that adrenalectomy may be effective in improving blood pressure in hypertensive patients with adrenal incidentalomas and MACS [15, 16]. Ongoing studies will provide more data on the efficacy of surgical (NCT02364089) [37] and medical treatment with steroidogenesis inhibitors (EudraCT: 2019–002008-41) [38] on hypertension in MACS (a full list of trials is available at clinicaltrials.gov).

The main limitation of this study is that the results were based on a self-reported survey, without a through revision of the data provided by each centre. However, this approach is appropriate to understand the current management of hypercortisolism among patients with hypertension on a large scale. Moreover, we did not investigate the referral rate to hypertensive centres from general practitioners. Indeed, the strength of this study are the large number of centres involved, spanning across the country, with a well-balanced representation of excellence and non-excellence centres.

## Conclusions

The current screening of hypercortisolism among hypertensive patients is still unsatisfactory. The results of this study provides a basis for planning future strategies to improve the screening rate of this condition by increasing the knowledge about the relationship between hypercortisolism and hypertension, and tailoring the choice of more appropriate screening tools based on the clinical setting (clinical suspicion of Cushing’s syndrome or adrenal incidentalomas), convenience for patients and physicians, and accuracy. These strategies should be targeted according to the different medical specialties and the type of centre.

## Supplementary Information

Below is the link to the electronic supplementary material.

Supplementary file1 (DOCX 29 KB)

## Data Availability

Data are available on request.
