# Supplementary material for: Screening of hypercortisolism among patients with hypertension: an Italian nationwide survey
Source: J Endocrinol Invest. 2024 Jun 24;47(12):3029–38. doi: 10.1007/s40618-024-02387-2 (PMC11549160; doi:10.1007/s40618-024-02387-2)
Supplement: Supplementary file 1 — Supplementary file1 (DOCX 29 KB) [file 40618_2024_2387_MOESM1_ESM.docx]

**SUPPLEMENTARY MATERIAL**

**Supplementary Table 1. Questionnaire.**

| **Survey on Management of Hypercortisolism and Cushing’s Syndrome** |
| --- |
| *(1) What is your specialty?*   1. Internal Medicine 2. Nephrology 3. Cardiology 4. Endocrinology 5. Other, specify |
| *(2) What is the average number of patients with hypertension seen in your service/centre in 1 year?* |
| *(3) How many patients are seen as first visit?* |
| *(4) What is the percentage of patients with resistant hypertension seen in your service/centre?* |
| *(5) How many cases of Cushing syndrome were diagnosed in the last 5 years in your service/centre?*  *(of which Pituitary N=……………; Adrenal N=………………….; other N=………………)* |
| *(6) In which group of patients, you request the evaluation of 24h urinary free cortisol or the overnight 1-mg dexamethasone suppression test if not performed before?*   1. Patients with hypertension and specific features (buffalo hump, moon facies, purple reddish striae) 2. All patients with diabetes and hypertension 3. All patients with hypertension and adrenal mass 4. All patients with hypertension and obesity 5. All patients with hypertension and obesity and adrenal mass 6. All patients with resistant hypertension 7. All patients with hypertension aged less than 50 years 8. When the phenotype is suspect, I refer the patient to an endocrinologist for further investigations |
| *(7) Which test(s) do you perform for screening of hypercortisolism?*   1. 1-mg overnight dexamethasone suppression test 2. 24h urinary free cortisol 3. Late night salivary cortisol 4. Basal cortisol and ACTH 5. Adrenal CT scanning or MRI 6. I refer to the endocrinologist without performance of any test |
| *(8) Are you aware of a referral centre in your area with expertise of Cushing syndrome management?* |
| *(9) How do you consider a patient with a level of cortisol of 2.5 μg/dL at 8 a.m. after overnight 1-mg dexamethasone suppression test?*   1. Positive 2. Negative 3. Grey zone, I request further tests (please describe) 4. I refer to an endocrinologist |
| *(10) How many patients with subclinical hypercortisolism (mild autonomous cortisol secretion) did you diagnose in the last 5 years?* |

The table reports the 10-items questionnaire designed for physicians treating patients with hypertension to investigate current screening methods for hypercortisolism.

**Supplementary Table 2. Number of patients according to geographical area, prevalent medical specialty, and excellence centers.**

| **Geographical Areas** | **North** (n=52) | | **Centre-South** (n=30) | | ***P-*value** |
| --- | --- | --- | --- | --- | --- |
| Average number of referred patient in 1 year (n) | 600 [300; 1338] | | 675 [300; 2000] | | 0.585 |
| Patients evaluated as first visit (n) | 175 [100; 300] | | 175 [50; 500] | | 0.828 |
| Patients with resistant hypertension (%) | 10.0 [5.0; 28.8] | | 12.5 [5.0; 20.0] | | 0.919 |
| Cases of Cushing syndrome in the last 5 years (n) | 1 [0; 4] | | 2 [ 0; 6] | | 0.658 |
| Cases of MACS in the last 5 years (n) | 1 [0; 4] | | 0 [0; 3] | | 0.207 |
| **Prevalent Specialty** | **Internal Medicine**  (n=36) | **Cardiology**  (n=25) | | **Others***  (n=21) | ***P-*value** |
| Average number of referred patient in 1 year (n) | 600 [300; 1876] | 800 [300; 1425] | | 500 [175; 1650] | 0.731 |
| Patients evaluated as first visit (n) | 225 [105; 463] | 150 [80; 350] | | 100 [30; 275] | 0.064 |
| Patients with resistant hypertension (%) | 12.5 [6.3; 30.0] | 10.0 [5.0; 12.5] | | 15.0 [10.0; 45.0] | **0.048** |
| Cases of Cushing syndrome in the last 5 years (n) | 3 [1; 7] | 0 [0; 2] | | 1 [0; 5] | **0.009** |
| Cases of MACS in the last 5 years (n) | 1 [0; 10] | 0 [0; 2] | | 0 [0; 4] | 0.222 |
| **Excellence Centres** | **Yes** (n=25) | | **No** (n=57) | | ***P-*value** |
| Average number of referred patient in 1 year (n) | 1500 [500; 2000] | | 450 [200; 1000] | | **<0.001** |
| Patients evaluated as first visit (n) | 300 [175; 650] | | 100 [60; 300] | | **<0.001** |
| Patients with resistant hypertension (%) | 15.0 [6.5; 30.0] | | 10.0 [5.0; 22.5] | | 0.895 |
| Cases of Cushing syndrome in the last 5 years (n) | 3 [1; 9] | | 0 [0; 3] | | **0.001** |
| Cases of MACS in the last 5 years (n) | 2 [1; 15] | | 0 [0; 2] | | **<0.001** |

The table reports number of referred patients with Cushing syndrome or subclinical hypercortisolism (MACS) after stratification for geographical areas, prevalent medical specialty, and type of centre. Data are reported as median [interquartile range]. *Others: endocrinology, nephrology, geriatrics. *P-*value < 0.05 were considered significant and highlighted in bold.

**Supplementary Table 3. Management of hypercortisolism and Cushing’s syndrome according to geographical areas.**

| **Geographical Areas** | **North**  (n=52) | **Centre-South**  (n=30) | ***P-*value** |
| --- | --- | --- | --- |
| *(6) In which group of patients, you request the evaluation of 24h urinary free cortisol or the overnight 1-mg dexamethasone suppression test if not performed before?* | | | |
| 1. Patients with hypertension and specific features 2. All patients with diabetes and hypertension 3. All patients with hypertension and adrenal mass 4. All patients with hypertension and obesity 5. All patients with hypertension and obesity and adrenal mass 6. All patients with resistant hypertension 7. All patients with hypertension aged less than 50 years 8. When the phenotype is suspect, I refer the patient to an endocrinologist | 40 (76.9)  4 (7.7)  32 (61.5)  9 (17.3)  19 (36.5)  30 (57.7)  18 (34.6)  10 (19.2) | 23 (76.7)  2 (6.7)  23 (76.7)  0 (0.0)  12 (40.0)  20 (66.7)  8 (26.7)  3 (10.0) | 1.000  1.000  0.160  **0.023**  0.752  0.424  0.454  0.356 |
| *(7) Which test(s) do you perform for screening of hypercortisolism?* | | | |
| 1. 1-mg overnight dexamethasone suppression test 2. 24h urinary free cortisol 3. Late night salivary cortisol 4. Basal cortisol and ACTH 5. Adrenal CT scanning or MRI 6. I refer to the endocrinologist without performance of any test | 28 (53.8)  30 (57.7)  6 (11.5)  24 (46.2)  6 (11.5)  7 (13.5) | 12 (40.0)  24 (80.0)  3 (10.0)  20 (66.7)  4 (13.3)  3 (10.0) | 0.227  0.054  1.000  0.073  1.000  0.739 |
| *(8) Are you aware of a referral centre in your area with expertise of Cushing syndrome management?* | | | |
| 1. Yes 2. No | 35 (67.3)  17 (32.7) | 22 (73.3)  8 (26.7) | 0.566 |
| *(9) How do you consider a patient with a level of cortisol of 2.5 μg/dL at 8 a.m. after overnight 1-mg dexamethasone suppression test?* | | | |
| 1. Positive 2. Negative 3. Grey zone, I request further tests 4. I refer to an endocrinologist | 8 (15.4)  0 (0.0)  22 (42.3)  22 (42.3) | 4 (13.3)  4 (13.3)  11 (36.7)  11 (36.7) | 1.000  **0.016**  0.617  0.617 |

Responses to questions 6-to-9 of the questionnaire after stratification for geographical areas. Data are reported as absolute numbers and frequencies, as appropriate. *P-*value < 0.05 were considered significant and highlighted in bold.

**Supplementary Table 4. Management of hypercortisolism and Cushing’s syndrome according to type of centre (excellent vs non excellent).**

| **Excellence Centres** | **Excellent Centres**  (n=25) | **Non Excellent Centres**  (n=57) | ***P-*value** |
| --- | --- | --- | --- |
| *(6) In which group of patients, you request the evaluation of 24h urinary free cortisol or the overnight 1-mg dexamethasone suppression test if not performed before?* | | | |
| 1. Patients with hypertension and specific features 2. All patients with diabetes and hypertension 3. All patients with hypertension and adrenal mass 4. All patients with hypertension and obesity 5. All patients with hypertension and obesity and adrenal mass 6. All patients with resistant hypertension 7. All patients with hypertension aged less than 50 years 8. When the phenotype is suspect, I refer the patient to an endocrinologist | 20 (80.0)  3 (12.0)  20 (80.0)  3 (12.0)  11 (44.0)  17 (68.0)  11 (44.0)  3 (12.0) | 43 (75.4)  3 (5.3)  35 (61.4)  6 (10.5)  20 (35.1)  33 (57.9)  15 (26.3)  10 (17.5) | 0.655  0.363  0.107  1.000  0.442  0.386  0.113  0.745 |
| *(7) Which test(s) do you perform for screening of hypercortisolism?* | | | |
| 1. 1-mg overnight dexamethasone suppression test 2. 24h urinary free cortisol 3. Late night salivary cortisol 4. Basal cortisol and ACTH 5. Adrenal CT scanning or MRI 6. I refer to the endocrinologist without performance of any test | 13 (52.0)  19 (76.0)  3 (12.0)  14 (56.0)  3 (12.0)  2 (8.0) | 27 (47.4)  35 (61.4)  6 (10.5)  30 (52.6)  7 (12.3)  8 (14.0) | 0.699  0.052  1.000  0.777  1.000  0.501 |
| *(8) Are you aware of a referral centre in your area with expertise of Cushing syndrome management?* | | | |
| 1. Yes 2. No | 25 (100.0)  0 (0.0) | 32 (56.1)  25 (43.9) | **<0.001** |
| *(9) How do you consider a patient with a level of cortisol of 2.5 μg/dL at 8 a.m. after overnight 1-mg dexamethasone suppression test?* | | | |
| 1. Positive 2. Negative 3. Grey zone, I request further tests 4. I refer to an endocrinologist | 6 (24.0)  1 (4.0)  13 (52.0)  5 (20.0) | 6 (10.5)  3 (5.3)  20 (35.1)  28 (49.1) | 0.172  1.000  0.150  **0.013** |

Responses to questions 6-to-9 of the questionnaire after stratification according to type of centre. Data are reported as absolute numbers and frequencies, as appropriate. *P-*value < 0.05 were considered significant and highlighted in bold.
